# Eat, flee, freeze: Division of labor in the larval zebrafish visuomotor system

**DOI:** 10.1073/pnas.2506018122

**Published:** 2025-05-05

**Authors:** Qing Wang, Herwig Baier

**Affiliations:** ^a^Department of Genes–Circuits–Behavior, Max Planck Institute for Biological Intelligence, Martinsried 82152, Germany

Imagine that as you are taking a walk in a park one day, you spot a round object hurtling toward you. Your brain quickly assesses the visual characteristics of this object and instructs your limbs to catch the errant football that has escaped its goal. Had the approaching object been much larger and faster, however, your brain might have interpreted it as an imminent threat, sending out instructions to coordinate a fleeing response in your body instead. Within fractions of a second, the brain is able to evaluate sensory stimuli and generate the appropriate motor instructions required to perform a desired behavior. This central question of how and where the brain flexibly transforms different visual stimuli into distinct motor behaviors is tackled here by Zhao and Tong et al. (1)

**Fig. 1.**
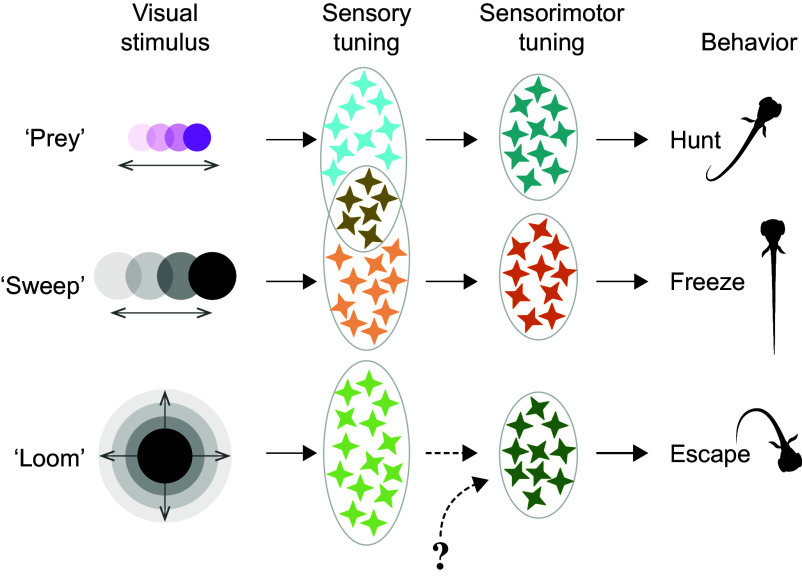
Schematic illustrating the three types of visual stimuli and the corresponding visually evoked behaviors explored by Zhao and Tong et al. “Prey” was simulated with a small, horizontally moving UV spot, which elicited hunting behavior characterized by eye convergence and J-shaped swim bouts. A larger horizontally moving spot (“sweep”) approximating a passing threat evoked freezing behavior characterized by immobility and bradycardia. Finally, a rapidly expanding “loom” stimulus imitating an approaching predator elicited large-amplitude escape swims. “Sensory neurons” (light-colored stars) responding robustly to visual stimuli were broadly tuned due to the substantial overlap in neurons that responded to both prey (light blue) and sweep stimuli (light orange). Behavior-correlated “sensorimotor (SM) neurons” (dark-colored stars) were highly specific to each evoked behavior. SM neurons corresponding to each behavior were spatially segregated in the brain and likely received visual inputs from their sensory neuron counterparts, except for the escape SM neurons. Escape SM neurons may not be connected to loom-sensitive sensory neurons, instead receiving inputs from other pathways in the brain. Illustration by Julia Kuhl and Herwig Baier.

Zhao and Tong et al. rely on the fact that simple visual stimuli can evoke complex naturalistic behaviors including hunting (2–4), fleeing (5–7), and freezing (this paper) in larval zebrafish. While the circuits that process visual stimuli have been extensively studied (8), observation of distinct visually evoked behaviors with simultaneous large-scale acquisition of neuronal activity data promises to reveal insights into the neural substrate of sensorimotor transformations. The zebrafish model further benefits from the considerable corpus of works that have identified the kinematically distinct features of various swimming behaviors (9, 10). Using volumetric two-photon microscopy, the authors were able to image around half of the larval zebrafish brain volume at single-cell resolution, obtaining a dataset comprising activity traces of over 60,000 active neurons across seven animals. Furthermore, by semi-immobilizing larvae in an agarose gel, the authors could concurrently perform high-speed recordings of the heart rate, eye movements, and tail movements in response to visual stimuli specifically optimized to evoke hunting, escape, and freezing behaviors (Fig. 1).

Although studies have been conducted on visually evoked hunting and escape behaviors in larval zebrafish, Zhao and Tong et al. are the first to characterize visually evoked freezing in detail. Similar to how a moving silhouette may evoke freezing behaviors in mice and flies due to its resemblance to a predator flying overhead, the authors discovered a dark, sweeping disc moving horizontally across the frontal visual field of the larvae while staying at a constant size of 15° in visual angle (“sweep stimulus”) could trigger immobility. The authors further observed a consistent drop of the heart rate of larval zebrafish during the stimulus window, showing how bradycardia could be another reliable measure of freezing behavior along with immobility. In addition to the sweep stimulus, the authors used other well-calibrated visual stimuli: a small (4°), horizontally moving UV-bright dot that mimicked prey to elicit hunting behavior (“prey stimulus”) and a large, rapidly expanding dark spot (6° to 60°) that mimicked a fast-approaching predator to elicit escape behavior (“loom stimulus”).

The minimal circuit components required for a visuomotor transformation in the brain include neurons that can process visual information and neurons that can integrate this information to produce an appropriate motor command. The authors reasoned that the former sensory-correlated neurons (or “sensory neurons”) should respond reliably to every instance of stimulus presentation, regardless of behavioral outcome. On the other hand, since animals do not behave in the same manner, if at all, at each stimulus exposure, the latter “SM neurons” should not only integrate sensory information but also other inputs that could influence the likelihood of performing certain behaviors, such as inputs that are informative of the state of the animal (11, 12). Thus, SM neuron activity should be more strongly correlated with behavioral onset. The authors made use of these first principles to classify the sensory and SM neurons associated with each visually evoked behavior by devising measures such as the sensory index (SI) and motor surplus index (MSI) for each neuron (13). They found that neurons scoring highly on SI were exceptionally regular in their responses to each stimulus presentation (sensory neurons). Though sensory neurons responded robustly to visual stimuli, sweep-sensitive and prey-sensitive sensory neurons were surprisingly broadly tuned and highly overlapping, with a large proportion of these neurons responding to both sweep and prey stimuli. By contrast, loom sensory neurons were more specific for the looming stimulus. Neurons that scored highly on MSI responded only in trials where a specific behavior was observed (SM neurons). These putative SM neurons with a high MSI typically also scored low on SI, and vice versa, indicating that sensory and SM neurons were indeed two functionally distinct neuronal populations that were specifically correlated with each stimulus/behavior pair.

Although studies have been conducted on visually-evoked hunting and escape behaviors in larval zebrafish, Zhao and Tong et al. are the first to characterize visually-evoked freezing in detail.

The authors further ascertained where these neurons were located in the brain with reference to the mapzebrain zebrafish atlas (https://mapzebrain.org/). While sensory neurons were primarily located in the optic tectum (OT), SM neurons corresponding to each behavior were distributed across different brain areas including the OT, pretectum, thalamus, and nucleus isthmi. The OT, homologous to the mammalian superior colliculus, merits scrutiny as a likely site of visuomotor transform. As the major visual processing hub of the zebrafish brain, it not only contains a retinotopic map of the visual scene (14, 15) where other sensory systems and state-dependent inputs are brought in conjunction (11, 16, 17) but is also known to send projections to premotor areas to facilitate goal-directed movements, including orienting and evasive behaviors (4, 18, 19). Within the OT, the authors discovered spatially segregated populations of hunting, freezing, and escape SM neurons that occupied the anterior, middle, and posterior OT, respectively. Moreover, the authors revealed that SM neurons for each behavior remained anatomically segregated in areas downstream of the OT. This indicates that the distinct behavioral outcomes have probably already diverged at the level of the OT and continue this way in the next processing stages.

While examining the positions of sensory neurons in the OT, the authors found that sweep and prey sensory neurons were intermingled with their SM counterparts, while loom sensory neurons showed a curious spatial separation from their escape SM partners. Given the close proximity of some of these neurons, connections between the sensory and SM neurons can be plausibly expected. However, it is also possible that SM neurons are only indirectly receiving visual information via other pathways in the brain that are also correlated with the behavioral outcome. To examine the possible connections between sensory and SM neurons, the authors removed the influence of neuronal activity related to behavioral outcome from the activity traces of sensory and SM neurons. They found that about a third of the sweep/freezing and prey/hunting neurons retained high correlations with one another, implying that a substantial number of freezing and hunting SM neurons were integrating visual information from their sweep and prey sensory neuron partners. Perhaps linked to the anatomical separation of loom sensory neurons and escape SM neurons is the fact that the authors could not find significant partial correlations between them, further suggesting that loom sensory neurons and escape SM neurons were not connected. It may be that these escape SM neurons are not the sites of visuomotor integration but are correlated with motor behavior via indirect circuits. The OT projects to motor control targets such as the reticulospinal neurons (4, 18, 19), one of which may include the pair of giant Mauthner cells that are responsible for triggering fast, ballistic escapes (20). Given the need for escape behavior to be rapid, loom-sensitive sensory neurons could potentially convey sensory drive to the Mauthner cell directly to induce low latency escapes, bypassing the escape SM neurons altogether.

Beyond the impressive technical prowess on display, Zhao and Tong et al. demonstrate here the versatility of large-scale brain imaging and behavioral analysis in the larval zebrafish model, be it from the perspective of sensory processing or behavior generation. One instance of this is their ability to use their imaging and refined correlation-based methods to identify SM neurons clustered in the vicinity of the AF7 pretectum. Previous work has shown that the AF7 pretectum of the larval zebrafish contains command neurons that drive hunting behavior when stimulated optogenetically but are notably unresponsive to visual prey stimuli, thus functioning very similarly to the SM neurons described here (21). The finding of these SM pretectal neurons also helps to answer one of the more intriguing questions posed by the authors: How could overlapping prey and sweep sensory neurons responsive to both types of visual stimuli still drive divergent hunting and freezing SM populations and vastly different behavioral outcomes? The authors suggest that these pretectal SM neurons could provide additional prey-related information to the tectal hunting SM neurons. This was further corroborated when partial correlation analysis revealed that a group of pretectal hunting SM neurons were indeed highly correlated with tectal hunting SM neurons.

While the authors’ approach does not prove causality, it nonetheless can identify functionally and anatomically distinct neuronal populations, reveal interactions between neuronal activity and behavior during sensorimotor transformations, and localize the potential sites of visuomotor transformations in the brain. This work comes at an exciting time in zebrafish neuroscience. With the rising popularity of connectomics and single-cell transcriptomics (22, 23), we anticipate this work to be a springboard for future investigations into the molecular identities, morphologies, and synaptic partners of neurons in visuomotor circuits. Some day in the not-so-distant future, we may even be able to pinpoint neural circuits in our own brains that decide, within a split second, whether to catch that approaching football, duck, or run away from it.
